# Pyrosequencing assessment of prokaryotic and eukaryotic diversity in biofilm communities from a French river

**DOI:** 10.1002/mbo3.80

**Published:** 2013-03-20

**Authors:** Geneviève Bricheux, Loïc Morin, Gwenaël Le Moal, Gérard Coffe, Damien Balestrino, Nicolas Charbonnel, Jacques Bohatier, Christiane Forestier

**Affiliations:** 1 Laboratoire “Microorganismes: Génome et Environnement”, Clermont Université, Université Blaise PascalBP 10448, F-63000, Clermont-Ferrand, France; 2 Laboratoire “Microorganismes: Génome et Environnement”, Clermont Université, Université d'AuvergneBP 10448, F-63000, Clermont-Ferrand, France; 3 CNRS, UMR 6023LMGE, F-63177, Aubiere, France; 4 Institut de Génétique et Microbiologie, UMR, CNRS8621 PRES, Paris Sud, France

**Keywords:** Aquatic communities, biofilm, pyrosequencing

## Abstract

Despite the recent and significant increase in the study of aquatic microbial communities, little is known about the microbial diversity of complex ecosystems such as running waters. This study investigated the biodiversity of biofilm communities formed in a river with 454 Sequencing™. This river has the particularity of integrating both organic and microbiological pollution, as receiver of agricultural pollution in its upstream catchment area and urban pollution through discharges of the wastewater treatment plant of the town of Billom. Different regions of the small subunit (SSU) ribosomal RNA gene were targeted using nine pairs of primers, either universal or specific for bacteria, eukarya, or archaea. Our aim was to characterize the widest range of rDNA sequences using different sets of polymerase chain reaction (PCR) primers. A first look at reads abundance revealed that a large majority (47–48%) were rare sequences (<5 copies). Prokaryotic phyla represented the species richness, and eukaryotic phyla accounted for a small part. Among the prokaryotic phyla, Proteobacteria (beta and alpha) predominated, followed by Bacteroidetes together with a large number of nonaffiliated bacterial sequences. Bacillariophyta plastids were abundant. The remaining bacterial phyla, Verrucomicrobia and Cyanobacteria, made up the rest of the bulk biodiversity. The most abundant eukaryotic phyla were annelid worms, followed by Diatoms, and Chlorophytes. These latter phyla attest to the abundance of plastids and the importance of photosynthetic activity for the biofilm. These findings highlight the existence and plasticity of multiple trophic levels within these complex biological systems.

## Introduction

Current knowledge of the microbial communities in rivers is scarce whereas these communities are hot spots of exchanges between bacteria from human sources and aquatic ecosystems (Vilanova et al. 2004; Martins da Costa et al. 2006; Moura et al. 2007; Galvin et al. 2010) and are natural receptacles for pollutant molecules from waste water treatment plants (organic and microbiological pollution), individual industries (chemical pollution), or agricultural activity (pesticides) (Brown and van Beinum 2009). Many studies on the impact of pollutants have been made on free microbial communities, seldom assessing the effects on the periphyton compartment. In the lotic environment, autotrophic (algae and cyanobacteria) and heterotrophic (bacteria, fungi, and protozoa) communities are mainly present as biofilms. Owing to their benthic nature, these communities are much more exposed than plankton communities. They can therefore provide more information about the effects of pollution and are excellent models for observing the health of rivers, because of their high sensitivity to environmental changes, their short life cycle, their diversity, and their ubiquity in aquatic environments (Lavoie et al. 2004).

These communities cannot be adequately studied by traditional culture-based techniques. As a result, a variety of molecular fingerprinting methods have been developed to assess the diversity of microbial communities such as ribosomal spacer analysis, terminal restriction fragment length polymorphism (t-RFLP), denaturing gradient gel electrophoresis (DGGE), 16S rRNA clone libraries, or fluorescence in situ hybridization (FISH) (Nocker et al. 2007; Rajendhran and Gunasekaran 2011).

The results obtained with these methods are likely to be incomplete as they do not capture the whole complexity of the communities and mostly address the bacterial compartment of the biofilms. High-throughput next-generation sequencing, such as 454 pyrosequencing (Margulies et al. 2005) of the small subunit (SSU) rRNA genes, offers an alternative, in which detailed community structure can be achieved in combination with fairly high taxonomic resolution (Sogin et al. 2006; Andersson et al. 2008). This approach has so far been used to explore human microbiomes (Costello et al. 2009; Turnbaugh et al. 2009; Koren et al. 2010; Lazarevic et al. 2010; Ling et al. 2010) and environmental samples such as soil (Roesch et al. 2007; Lauber et al. 2009) and oceans (Sogin et al. 2006; Qian et al. 2010). A few investigations have been performed on environmental water samples from either waste or drinking water (Hong et al. 2010; Lee et al. 2010; Liu et al. 2012). Studies on biofilms have demonstrated the preponderance of bacteria affiliated to the subclass of beta-proteobacteria and Cytophaga-Flavobacterium in bacterial communities in anthropized environments characterized by high pollution (organic and metallic) (Brummer et al. 2000, 2003). Recently, Besemer demonstrated the predominance of beta-proteobacteria in biofilms and suspended communities in water streams, as well as an overall lower diversity in the biofilm communities compared with those in suspended stream water (Besemer et al. 2012). Eukaryotes are known to be integrated inside natural aquatic biofilms, but available biodiversity data are poorly documented (Dorigo et al. 2007, 2010; Fechner et al. 2010).

In this study, we used extensive 454 pyrosequencing analysis of different regions of SSU rRNA genes of biofilm communities formed in the flow of a small river receiving urban effluent from a wastewater treatment plant. To characterize the largest range of rDNA sequences, we used a set of nine couples of primers hybridizing to different regions of the SSU rRNA genes. Eukaryotes being closely associated with bacteria in these communities, the eukaryotic population was also investigated.

## Materials and Methods

### Biofilm sampling

Biofilms were formed on several glass substrates, which were placed nearby within the stream of the Jauron River, and incubated for 19 days in April 2009. Jauron is a small river located downstream of the wastewater effluent discharge of Billom, a town of about 5000 inhabitants, 25 kms south of Clermont-Ferrand, France (GPS location: 45°44′19.36″N, 3°20′20.903E, 340 m).

### Microscopic observations

Biofilms were rinsed with cacodylate buffer, fixed for 60 min with a solution of 4% glutaraldehyde with 0.15% ruthenium red (in 0.2 mol/L cacodylate buffer), rinsed for 5–10 min in 0.2 mol/L cacodylate buffer, and then postfixed in 1% OsO4 (in 0.1 mol/L cacodylate buffer) for 1 h. They were then transferred directly to cold 50% ethanol and dehydrated through a series of increasing concentrations of cold ethanol. After drying, the samples were mounted on stubs and coated with gold in a sputter coater and observed with a Jeol 6060-low vacuum microscope.

### DNA extraction, PCRs, and pyrosequencing

Biofilm samples were collected from the surface of several glass substrate slides scattered across the sampling place. The biofilms formed at the surface of the slides after 19 days of incubation were mixed and resuspended in mineral water by vortexing and sonication. The resulting biofilm suspension was then centrifuged to collect the pellets. The DNA was extracted according to a chemical and enzymatic DNA extraction protocol using the Macherey-Nagel Nucleospin tissue kit after mechanical disruption of the cells by a Precellys® apparatus (Bertin Technologies, Montigny le Bretonneux, France). The bulk community DNA sequences were amplified with nine pairs of primers (Table 1) covering the different regions of the SSU ribosomal genes: two pairs of universal primers were used for both eukaryotes and prokaryotes (U1 and U2), one pair was used to detect archaea (A), four pairs were specific for prokaryotes (B1, B2, B3, and B4), and two pairs were specific for eukaryotes (Euc1 and Euc2). Primers were chosen according to previous analyses showing that biodiversity richness is linked to the primer sequences (Medlin et al. 1988; Reysenbach et al. 1994; Dassarma and Fleischmann 1995; Vetriani et al. 1999; Baker et al. 2003; Huse et al. 2008). Only one primer was adapted for this study (343FB) (Hansen et al. 1998).

**Table 1:** Sequences of primers used for pyrosequencing

Primer name	Sequence (5′–3′)	Target	References
1053F	GCATGGCYGYCGTCAG	Universal SSU rRNA gene (U1)	Dassarma and Fleischmann 1995;
1510R	GGTTACCTTGTTACGACTT		Reysenbach et al. 1994;
1053F	GCATGGCYGYCGTCAG	Universal SSU rRNA gene (U2)	Dassarma and Fleischmann 1995;
1391R	GACGGGCGGTGWGTRCA		Reysenbach et al. 1994;
1098FA	GGCAACGAGCGMGACCC	Archaeal SSU rRNA gene (A)	Reysenbach et al. 1994;
1510R	GGTTACCTTGTTACGACTT		Reysenbach et al. 1994;
528FE	CGGTAATTCCAGCTCC	Eukaryotic SSU rRNA gene (Euc1)	Medlin et al. 1988;
1193RE	GGGCATMACDGACCTGTT		Medlin et al. 1988;
7FE	ACCTGGTTGATCCTGCCAG	Eukaryotic SSU rRNA gene (Euc2)	Vetriani et al. 1999;
529RE	ACCGCGGCKGCTGGC		Dassarma and Fleischmann 1995;
8FB	AGAGTTTGATCCTGGCTCAG	Bacterial SSU rRNA gene (B1)	Reysenbach et al. 1994;
534RB	ATTACCGCGGCTGCTGGC		Dassarma and Fleischmann 1995;
517FB	GCCAGCAGCCGCGGTAA	Bacterial SSU rRNA gene (B2)	Reysenbach et al. 1994;
1046RB	CGACAGCCATGCANCACCT		Huse et al. 2008;
343FB	TACGGRAGGCAGCAG	Bacterial SSU rRNA gene (B3)	This study
1046RB	CGACAGCCATGCANCACCT		Huse et al. 2008;
1099FB	GYAACGAGCGCAACCC	Bacterial SSU rRNA gene (B4)	Reysenbach et al. 1994;
1510R	GGTTACCTTGTTACGACTT		Reysenbach et al. 1994

Forward primers contained the Roche 454 pyrosequencing adapter FLX A plus MID (Multiplex identifier) and the specific designed RNA sequence, while the reverse primers contained the FLX B adapter plus the same MID plus the designed RNA sequence. Polymerase chain reactions (PCRs) were performed with 50 ng of template DNA and 20 pmol of each forward and reverse primer, 100 *μ*L of mix supplemented with 1.25 U of Phusion High-Fidelity DNA Polymerase Finnzyme (Thermo Scientific Fisher, Villebon-sur-Yvette, France), using a GeneAmp PCR system 2700 thermocycler (Applied Biosystems®, Life Technologies, Villebon-sur-Yvette, France). The two-step PCR conditions were as follows: 95°C for 1 min, 30 cycles of denaturation (95°C; 10 sec), annealing (60°C; 30 sec), and extension (72°C; 30 sec), followed by the final elongation (72°C; 10 min). The DNA was then quantified using a DNA quantitation kit fluorescence assay (Sigma-Aldrich, St Quentin Fallavier, France) and DNAs from the different reactions were mixed in equimolar proportions. Amplicons were then subjected to pyrosequencing using a Genome Sequencer 454 Titanium GS FLX (Eurofins, MWG/Operon, Germany).

### Pyrosequencing data analysis

#### PyroSuite

This program (available upon request) was developed to combine algorithms from several packages in order to (i) compute indices and rarefaction curves to determine whether the data set is representative of the sample biodiversity (http://www.mothur.org/); (ii) compare the sampled sequences against a local dedicated bank for automated identification (Blastn http://blast.ncbi.nlm.nih.gov or Blat http://genome.ucsc.edu); and (iii) study their clusterization in terms of relative abundance and annotated phylogenetic trees (Phylip (http://www.phylip.com/)).

PyroSuite includes several subroutines, of which the following are the two most important.

#### PyroSeqToAlignedSeq

This handles the Mothur Software (Schloss 2009), reading the commercial sequence fasta file, selecting a valid subset of the sequence stretches and producing an alignment fasta file through several algorithms described in the “Costello stool analysis” (Costello et al. 2009). Reads from commercial files were shortened when more than two undetermined bases (N) were found and Phred quality scores were *Q* ≤ 30. Sequences with more than eight repeats or smaller than 100 bp were eliminated, as well as primer sequences with mismatches. Sequence boundaries were chosen and SILVA (Pruesse et al. 2007) was used as the reference alignment in the process according to the analyzed amplicons data set (Bacteria, Archae, Eukarya, or Universal). The subroutine also computes rarefaction curves.

#### IdentifySeq

This subroutine uses different information sources: taxonomic files (GenBank), a dedicated annotated databank fasta file extracted from the updated archive of Genbank, EMBL, and DDBJ, and a subset of the alignment fasta file produced by PyroSeqToAlignedSeq. The sequences of this last file were processed with BLAST or BLAT against the annotated bank for identification, producing two files containing the BLAST result and ID of each sequence. Also produced are two files, which can identify and calculate the relative importance of redundant sequences.

## Results

### Microscopy analysis

**Figure 1:**
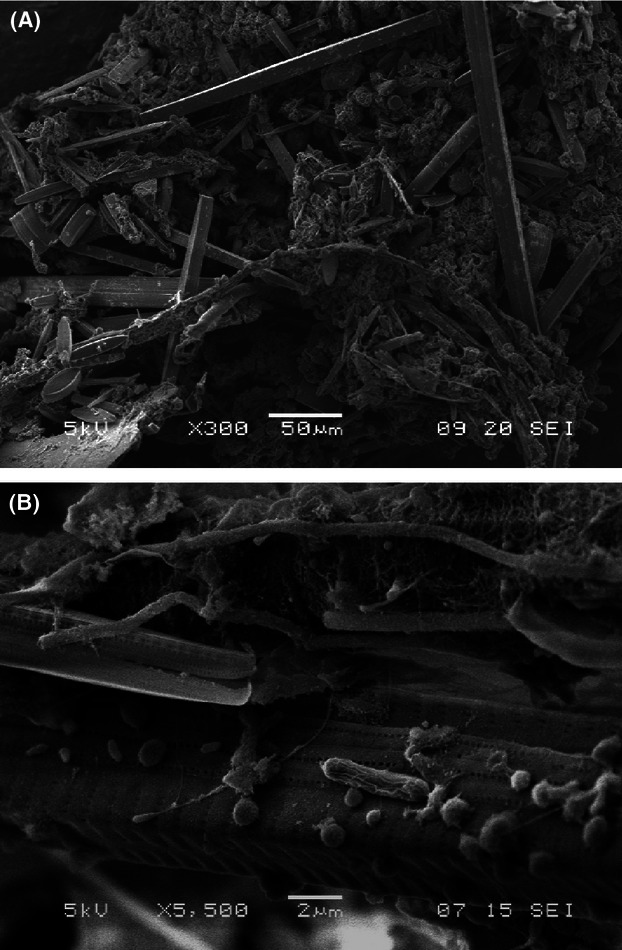
Scanning electron microscopy (SEM) images representative of biofilms grown for 19 days in riverine water on glass slides. (A) original magnification 300×, Bar = 50 *μ*m, (B) original magnification 5500×, Bar = 2 *μ*m.

Scanning electron microscopy observations performed with 19-day-old biofilm samples revealed heavily coated surfaces with a high number of diatoms at the interface between the biofilm and free running water (Fig. 1A). Rod-shaped bacteria were visible on the surface of diatoms (Fig. 1B) but most of these microorganisms were embedded in heavy extracellular material.

### 454 pyrosequencing strategy

The 454 pyrosequencing of biofilm DNA extract produced 26,423 raw reads. After the removal of 3994 low-quality reads (15.1%), 21,998 reads with a read length of 400 ± 50 bp (mean ± standard deviation) were used for further analyses.

**Figure 2:**
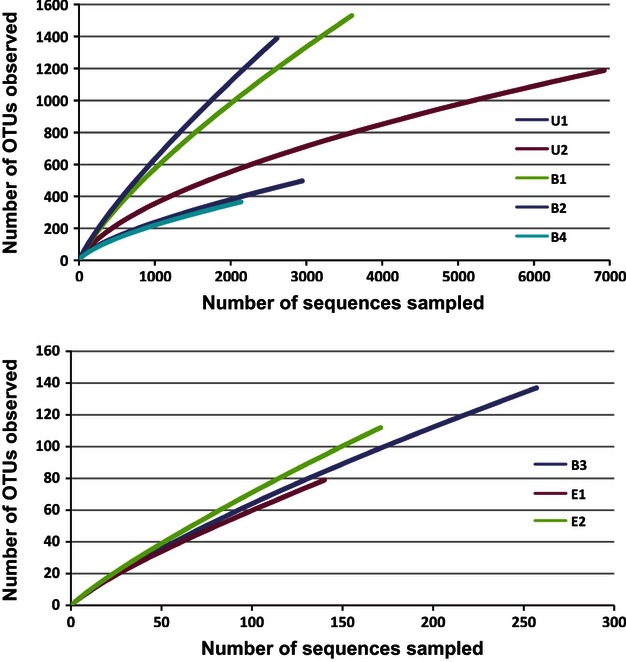
Rarefaction analysis of the amplicons obtained with the different pairs of primers at a level of 97% 16S rRNA similarity.

Rarefaction curves were produced for each pair of primers, with an operational taxonomic units (OTU) 3% species cutoff (Fig. 2). Two sets clearly appeared. In the first, the curves tended to be asymptotic and included U1, U2, and B4 primer pairs, while in the second OTU numbers increased with the sequence numbers (B1, B2, B3, Euc1, and Euc2 primer pairs), indicating that the sequencing effort was not sufficient in these cases. The second set was correlated with the weak number of sequences for B3, Euc1, and Euc2 primer pairs. The A primer pair was designed to recover archaeal sequences in the sample; sequence analysis of the resulting amplicons revealed the presence of bacterial sequences, to the exclusion of any archaeal sequence, indicating that a PCR mismatch annealing led to bacterial amplification for the forward archaeal specific primer. Further PCRs with different specific archaeal primers failed to recover any archaeal sequence (data not shown), thus confirming this result. The B3, Euc1, and Euc2 primer pairs were designed to recover about 700-bp-long amplicons. This length is the upper limit of the Titanium pyrosequencing technology, and may explain why the output of sequences was highly reduced. Likewise, as we made an equimolar mix of each amplicon, we can predicate that longer sequences were less numerous in the final mix.

Diversity indices, calculated with Mothur (Schloss 2009) are shown in Table 2. Evenness index was the highest and Coverage estimation the lowest for the B3 pair of primers, in correlation with the low sequence number and the growing rarefaction curves mentioned above. However, Euc1 and Euc2 pairs of primers displayed a medium evenness index and a good Coverage estimation, despite the low number of sequences recovered.

**Table 2:** Diversity and evenness indices of the SSU rDNA gene sequences from the pyrosequencing analysis according to the primers used

Primers	NS	Sobs (0.03)	ChaoI	Shannoneven	Shannon	Coverage
U1(V7–V9)	3058	284	642 (512;847)	0.63	3.58 (3.52;3.65)	0.95
U2(V7–V8)	7099	993	2163 (1917;2475)	0.71	4.91 (4.87;4.96)	0.92
Euc1(V4–V6)	192	37	125 (67;295)	0.60	2.16 (1.89;2.43)	0.84
Euc2(V1–V3)	258	71	184 (119;338)	0.78	3.31 (3.16;3.46)	0.87
B1(V1–V3)	5298	1523	3718 (3340;4175)	0.79	5.79 (5.74;5.85)	0.85
B2(V4–V6)	3287	846	2019 (1755;2361)	0.78	5.25 (5.19;5.31)	0.87
B3(V3–V6)	454	137	619 (389;1058)	0.86	4.22 (4.02;4.41)	0.58
B4(V7–V9)	2352	264	552 (449;713)	0.65	3.64 (3.56;3.73)	0.92
Total	21,998					

NS, number of sequences.

### Number of amplicons obtained according to the set of primers

**Figure 3:**
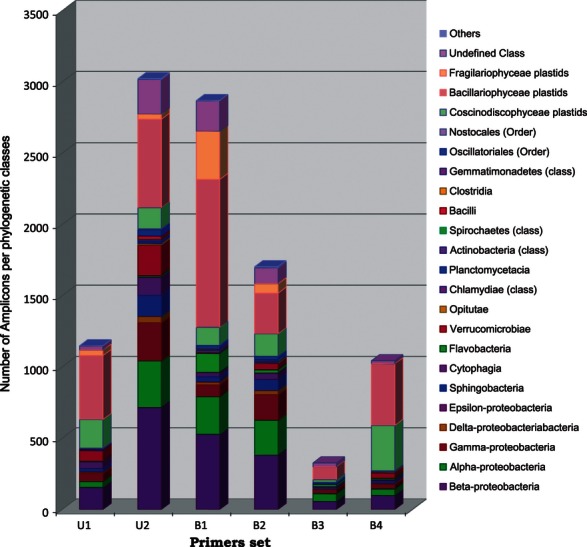
Taxonomic distribution of sequences assigned to prokarya according to primer sets.

Figure 3 displays the diversity of sequences at the class level in relation to the primer pair that was used. The two pairs of universal primers (U1 and U2) were localized at the end of the SSU ribosomal gene, involving the V7 and V8 domains plus the V9 domain for U1. The U2 primer pair (V7–V8) gave rise to about three times as many amplicons as the U1 primer pair (V7–V9). A proportion of 12.4% of the reads were classified as unidentified sequences for the U2 primer pair, and 5.7% for the U1 primer pair. Universal primers specifically recognized some taxa, such as Chlamydiae, Chloroflexi, and Chlorophycota plastids, but no eukaryotic sequence was detected. The four pairs of primers specific for bacteria (B1, B2, B3, and B4) recognized different variable domains of the molecule from V1 to V9. B1 primer pair (V1–V3) produced about twice as many amplicons as the B2 primer pair (V4–V6), about three times as many amplicons as the B4 primer pair (V7–V9), and about ten times as many amplicons as B3 primer pair (V3–V6). More than half of the B1 amplicons corresponded to the chloroplast sequences of diatoms. The B1 primer pair also produced more sequences than the universal primers among the Bacillariophyta chloroplasts. However, phyla such as Nitrospirae were recognized only by the B4 primer pair, while Deinococcus-Thermus was recognized only by the B2 primer pair. Primer pairs B3 and B4 revealed a weak diversity, dominated by chloroplastic sequences for primer pair B4. The B1 primer pair produced 11% unidentified sequences, as against 10.2%, 17%, and 4.5%, respectively, for primers B2, B3, and B4. The presence of eukaryotic sequences was confirmed by the simultaneous use of two eukaryote specific primers (Euc1 and Euc2). No member of the domain Archaea was detected, although 144 unique sequences were initially retrieved using primer pairs designed to detect archaea.

### Composition of the biofilm communities

After alignment of the 21,998 reads, 13,804 unique sequences derived from all pairs of primers were retained for OTU clustering and a final number of 4264 OTUs was obtained, on the basis of a 3% distance interval for species grouping (Table S1). These unique sequences were then grouped according to different taxonomic levels within each set of amplicons. Tables 3 and 4 show the result at the phylum and class levels. Whatever the primer used, Bacillariophyta plastids largely predominated, followed by Proteobacteria and Bacteroidetes. Verrucomicrobia were as abundant as Bacteroidetes when detected with universal and B4 primer pairs, but much less so with the B1 and B2 primer pairs, and were not detected with the B3 primer pair. Cyanobacteria, while not as abundant as the phyla cited above, were also significantly present. The other phyla were either poorly represented, or detected with few primer pairs.

**Table 3:** Table summarizing the distribution of each phylum according to the pair primers used for amplification

Phylum/primers	U1 (V7–V9)	U2 (V7–V8)	A (V7–V9)	B1 (V1–V3)	B2 (V4–V6)	B3 (V3–V6)	B4 (V7–V9)	Euc1	Euc2
Proteobacteria	267-23,24	1367-45,21	18-12,5	900-31,36	839-49,21	142-41,04	184-17,07		
Bacteroidetes	81-7,05	331-10,95		210-7,32	161-9,44	37-10,69	39-3,62		
Verrucomicrobia	77-6,70	247-8,17	1-0,69	5-0,17	54-3,17		38-3,53		
Cyanobacteria	9-0,78	50-1,65	2-1,39	26-0,91	24-1,41	10-2,89	10-0,93		
Firmicutes	5-0,44	24-0,79		6-0,21			4-0,37		
Actinobacteria	3-0,26	11-0,36		16-0,56	9-0,53	1-0,29	2-0,19		
Planctomycetes	1-0,09	10-0,33			6-0,35				
Chlamydiae		5-0,17							
Nitrospirae		2-0,07			2-0,12				
Spirochetes		4-0,13		2-0,07	2-0,12				
Acidobacteria					2-0,12	1-0,29			
Gemmatimonadetes		1-0,03		1-0,03	7-0,41	3-0,87			
Chloroflexi		2-0,07							
Deinococcus-Thermus					3-0,18				
Bacillariophyta plastids	675-58,75	793-26,22	123-85,42	1504-52,40	521-30,56	131-37,86	785-72,82		
Bacillariophyta								3-3,30	160-71,75
Euglenida plastids		5-0,17		1-0,03	3-0,18				
Chlorophycota plastids		3-0,10							
Chlorophycota								7-7,69	6-2,69
Eustigmatophyceae plastids					3-0,18				
Streptophyta plastids	1-0,09	3-0,10		2-0,07					
Annelida								69-75,82	49-21,97
Arthropoda								1-1,10	
Chytridiomycota								2-2,20	
Ascomycota								1-1,10	
Nemata								4-4,40	
Mollusca									10-0,45
Rotifera									1-0,45
Undefined Phylum	30-2,61	166-5,49		197-6,86	69-4,05	21-6,07	16-1,48	4-4,40	6-2,69
Total	1149	3024	144	2871	1705	346	1078	91	223

The composition of each phylum is given in term of number of different amplicons and percentage.

**Table 4:** Table summarizing the distribution of each class according to the pair primers used for amplification. The composition of each class is given in term of number of different amplicons with a confident score of >80%

Classes/primers	U1(V7–V9)	U2(V7–V8)	A (V7–V9)	B1(V1–V3)	B2(V4–V6)	B3(V3–V6)	B4(V7–V9)	Euc1	Euc2
Beta-proteobacteria	155-13,49	715-23,64	8-5,56	527-18,36	380-22,29	56-16,18	98-9,09		
Alpha-proteobacteria	41-3,57	327-10,81	7-4,86	264-9,20	246-14,43	55-15,90	45-4,17		
Gamma-proteobacteria	56-4,87	268-8,86	3-2,08	83-2,89	179-10,50	30-8,67	33-3,06		
Delta-proteobacteria	9-0,78	44-1,46		19-0,66	29-1,70		5-0,46		
Epsilon-proteobacteria	2-0,17			2-0,07			3-0,28		
Sphingobacteria	24-2,09	148-4,89		36-1,25	78-4,57	12-3,47	15-1,39		
Cytophagia	48-4,18	126-4,17		30-1,05	44-2,58	7-2,02	7-0,65		
Flavobacteria	4-0,35	11-0,36		134-4,67	23-1,35	16-4,62	11-1,02		
Verrucomicrobiae	73-6,35	216-7,14	1-0,69	5-0,17	45-2,64		38-3,53		
Opitutae		10-0,33			3-0,18				
Chlamydiae (class)		5-0,17							
Planctomycetacia	1-0,09	10-0,33			6-0,35				
Actinobacteria (class)	3-0,26	11-0,36		16-0,56	9-0,53	1-0,29	2-0,19		
Spirochetes (class)		4-0,13		2-0,07	2-0,12				
Bacilli	5-0,44	24-0,79		3-0,10			3-0,26		
Clostridia				3-0,10			1-0,09		
Gemmatimonadetes (class)		1-0,03		1-0,03	7-0,41	3-0,87			
Nitrospira (class)		2-0,07			2-0,12				
Acidobacteria (class)					2-0,12				
Spartobacteria					1-0,06				
Hadobacteria					3-0,18				
Oscillatoriales (order)	7-0,61	42-1,38	2-1,39	260,91	23-1,35	10-2,89	10-0,93		
Nostocales (order)	2-0,17	8-0,27			1-0,06				
Coscinodiscophyceae plastids	201-17,49	147-4,86	49-34,03	127-4,43	159-9,33	19-5,49	319-29,59		
Bacillariophyceae plastids	449-39,08	621-20,54	62-43,06	1038-36,17	285-16,72	89-25,72	432-40,07		
Bacillariophyceae								2-2,20	148-66,37
Fragilariophyceae plastids	38-3,31	36-1,19	12-8,33	338-11,78	66-3,87	19-5,49	34-3,15		
Fragilariophyceae								1-1,10	12-5,38
Conjugatophyceae plastids	1-0,09	1-0,03		2-0,07					
Trebouxiophyceae plastids		2-0,07							
Klebsormidiophyceae plastids		1-0,03							
Ulvophyceae plastids		1-0,03							
Ulvophyceae								6-6,59	5-2,24
Florideophyceae plastids					1-0,06				
Florideophyceae								1-1,10	1-0,45
Chrysomonada									1-0,45
Holotricha								1-1,01	
Phyllopharyngea								1-1,10	
Nassophorea									1-0,45
Litostomatea									1-0,45
Maxillopoda								1-1,10	
Chromadorea								2-2,20	
Chytridiomycetes								2-2,20	
Hemiascomycetes								1-1,10	
Ichthyosporea								1-1,10	
Polychaeta									1-0,45
Bivalvia									1-0,45
Monogononta									1-0,45
Clitellata								69-75,82	49-21,97
Undefined class	30-2,61	243-8,04		214-7,45	111-6,51	29-8,38	22-2,04	3-3,30	2-0,90

At the class level, Bacillariophyceae plastids prevail over Coscinodiscophyceae plastids, and largely over araphid pennate diatom plastids, except with the B1 primer pair for which araphid pennate diatom plastids dominate Coscinodiscophyceae plastids. Other plastid sequences were poorly represented. Among the proteobacteria, beta-proteobacteria largely predominate, followed by alpha- and gamma-proteobacteria; a few Delta-proteobacteria were also detected, but not with the B3 primer pair. Among the Bacteroidetes, Cytophagia and Sphingobacteria were the most common, while Flavobacteria were poorly abundant, except with the B1 primer pair for which they predominated over the other classes. Cyanobacteria were mainly Oscillatoriales (Phormidium). The most diversified eukaryotic phyla included Annelid worms and Stramenopiles, followed by Chlorophytes. The microalgae reflect the abundance of plastids and the importance of photosynthetic activity within biofilm. The remaining eukaryotic phyla were poorly diversified, but included a variety of protistan phyla-like ciliates, Cercozoa, Euglenozoa, and Foraminifera, but also fungi, nematodes, rotifers, arthropods, and mollusks.

**Figure 4:**
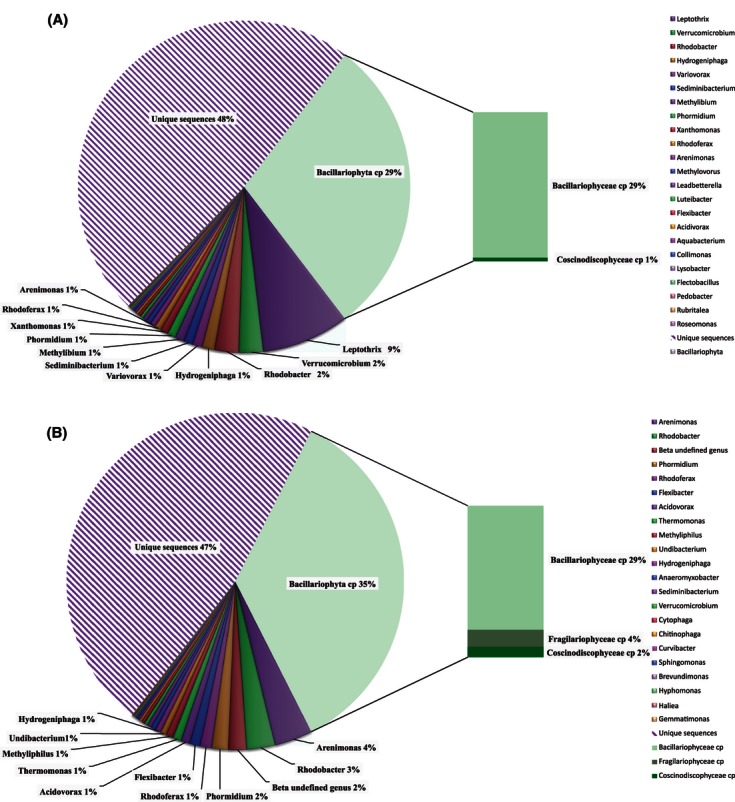
Taxonomic distribution and abundance observed with U2 and B2 primers (A and B, respectively). The composition and percentage of Bacillariophyta are shown in detail. Only results ≥1% are given.

Grouping sequences obtained with the U2 and B2 pairs of primers according to the abundance of each amplicon (i.e., including redundant sequences) are shown in Fig. 4A and B, respectively. Blast results were manually tested for species identifications when redundancy was significant (>5 identical amplicons). Both figures displayed a similar overall composition, with a very high number of unique sequences (47–48%), and a high proportion of Bacillariophyta plastid sequences (30–35%). Bacillariophyta plastid sequences included the three major diatom classes: a large majority (29%) of raphid pennate diatoms (Bacillariophyceae), few nonraphid pennate diatoms (Fragilariophyceae), and centric diatoms (Coscinodiscophyceae). Less abundant taxa included those that were detected with both U2 and B2 sets of primers, such as the filamentous phototrophic cyanobacteria Phormidium, and the genera Rhodobacter and Rhodoferax, for which most species are phototrophic. In contrast, Leptothrix, sheathed bacteria that are able to oxidize Fe (II) and Mn (II), and Verrucomicrobium were detected with the sole B2 primers while Arenimonas was obtained with the U2 primers.

The analysis of eukaryotic sequences obtained from the Euc2 primers showed a predominance of diatoms (66%), followed by annelids (23%). The remaining sequences were unique. Annelids predominated in data obtained from Euc1 primers (75%) (Table 3).

An overall analysis combining the information given by the different primer pairs was not possible because no single region of rDNA can differentiate among all bacteria or eukaryotic cells (Liu et al. 2008; Kim et al. 2011b).

## Discussion

Biofilms are spatially structured communities of microbes with a complex web of symbiotic interactions. Different studies pointed out a larger heterogeneity at the local scale (due to local aggregates, availability of nutrients, etc.) than at the global scale, for which larger samples are homogenized. In water, microbial populations are influenced by fluids circulation which may impact the local populations drastically, giving rise to a somewhat similar diversity/structure on the large and global scales and a much more divergent picture at the local scale. This study presents the analysis of a biofilm community formed on several glass slides placed into a single point of a river that was initially investigated by DGGE (Bricheux et al. 2013). Massive barcoded pyrosequencing was applied to perform a comprehensive analysis of the composition of the bacterial and eukaryotic communities of biofilms from this anthropized riverine water. This technology is a powerful method for investigating the diversity of complex ecosystems compared to conventional cloning analyses whereas not exempted of some bias or limitations (Tedersoo et al. 2010; Pinto and Raskin 2012). As every recent technologies, improvement will be done, notably with the arrival of ∼3000-base single-molecule reads of Pacific Biosciences (Eid et al. 2009; Ribeiro et al. 2012). To detect most of the organisms in this ecosystem, we used several primer pairs, either universal or specific for prokaryotic, eukaryotic, and archaeal cells, which targeted the ribosomal DNA gene region, owing to the richness of the corresponding databases. The number of sequences recovered by each primer pair was highly variable and illustrates the well-known amplification bias introduced by primer selection (Polz and Cavanaugh 1998; Baker et al. 2003; Baker and Cowan 2004; Acinas et al. 2005; Rajendhran and Gunasekaran 2011). Our results corroborate previous findings showing that bacterial V1–V3 domains better illustrate species richness and diversity (see Table 2 Chao1 and Shannon estimates) (Youssef et al. 2009; Kim et al. 2011a), and that a shorter amplicon produces greater richness (for example, U1 vs. U2) (Engelbrektson et al. 2010).

Bacterial and universal primer pairs gave rise to the highest number of sequences (6786–7567). In contrast, few amplicons were obtained with eukaryotic specific primers Euc1 and Euc2 (268–422, see Table 2), which could explain why no eukaryotic sequence was detected with universal primers. However, the Euc1 and Euc2 pairs of primers displayed a medium Shannon evenness index and a good Coverage estimation, despite the low number of sequences retrieved. It is likely therefore that the low sequence number reflects eukaryotic abundance in the sample and is not only related to the technical limitation due to the length of the amplicons.

Altogether, 4264 OTUs were detected, demonstrating the considerable diversity of anthropized riverine water biofilms, as observed in reports on other complex environments (Sogin et al. 2006; Huber et al. 2007; Roesch et al. 2007; Rusch et al. 2007; Andersson et al. 2009). Our results indicated a good diversity among the prokaryotes with a predominance (if diatom chloroplastic reads are excluded) of Proteobacteria (beta- and alpha-) and with Bacteroidetes and Verrucomicrobia as subdominant phyla. The same culture-independent technology has shown similar distributions in biofilm communities of water distribution systems, albeit with a much lower total number of OTUs (133 and 208) (Hong et al. 2010), and in wastewater treatment plant samples (Sanapareddy et al. 2009). Proteobacteria were also predominant in samples from an anoxic basin of a wastewater treatment plant (Chouari et al. 2010), from a drinking water treatment plant (Kwon et al. 2011), and from freshwater collected in a river (Vaz-Moreira et al. 2011). Comparison of these data with those of previous studies on river biofilms using different methods is awkward because of the different levels of resolution. However, using FISH and confocal laser scanning microscopy, Manz et al. (1999) showed that lotic biofilms were mostly composed in their initial formation steps of beta-Proteobacteria, followed by an increase in both alpha-proteobacteria and bacteria affiliated to the Cytophaga-Flavobacterium group. The predominance of beta-Proteobacteria and of the Cytophaga-Flavobacterium (former name of Bacteroidetes) cluster was also observed in biofilms formed in urban rivers as shown by FISH and DDGE analysis of 16S SSU ribosomal gene fragments (Brummer et al. 2000; Araya et al. 2003). Thus, the prevalence of Proteobacteria in anthropized riverine water biofilms could simply be the result of their predominance or could be related to the greater ability of these bacteria to initiate biofilm formation by adhering to surfaces. It is noteworthy that the most abundant bacterial groups detected by pyrosequencing in soil samples from several sites across the western hemisphere were also Proteobacteria and Bacteroidetes (Roesch et al. 2007). Interestingly, in biofilms of Swedish freshwater, beta-proteobacteria represent more than one third of the reads, followed by Flavobacteria (Besemer et al. 2012) whereas in our biofilm, the beta-proteobacteria were followed by the alpha-proteobacteria. Although we did not determine the bacterial composition of soil around our aquatic sampling sites, we can postulate that these wastewater effluent biofilms receive microorganisms both from the wastewater treatment plant (activated sludge) effluent and from the soil environment by water shedding. The presence in our samples of Rhodoferax, which are able to degrade phenoxy-herbicides (Ehrig et al. 1997), suggests that these communities could have been previously in contact with such pollutants. Potential human pathogens were also identified in all samples by culture (data not shown), mostly Enterobacteriaceae (Klebsiella and Enterobacter), Aeromonas, and Pseudomonas. Most of these isolates were resistant to antibiotics, with 75% of them producing cephalosporinases and 48% showing resistance to quinolones (data not shown). It is likely that sewage treatment processes are unable to avoid the dissemination of such resistant bacteria into the environment, resulting therefore in their contact with water- or soil-borne microorganisms.

In this study, no member of the domain Archaea was detected, although 144 sequences were initially retrieved using primer pairs designed to detect archaea. These amplicons actually corresponded mainly to bacterial sequences, predominantly plastids. One primer (1510R) from the A pair was not archaeal specific and could be responsible in part for this mispriming (Baker and Cowan 2004). Archaea have been shown to be present in significant amounts in some marine plankton, soil, lakes, and river (Schleper and Nicol 2010). However, our results are in agreement with the previous report of Manz et al. (1999) on lotic biofilms in which they used using FISH technology.

The main feature in the overall composition of the biofilms studied was a very high number of rare sequences (<5 copies, 46–47%), which is per se informative. This number is clearly linked to the sequencing effort and is affected by PCR bias, but the relative amplicon abundance was significant for the overall biofilm composition. Rare sequences could represent microorganisms that are not physiologically active or having only a minor influence on biofilm physiology. However, it has been shown that a relatively low-abundance organism (around 0.3% of all cells) can be responsible for significant fractions of metabolite fluxes (Musat et al. 2008). Besemer et al. (2012) compared the rank-abundance curves of bulk and active communities with stream water biofilms and reported that they were indistinguishable. As these communities are composed of a few dominant OTUs and a large fraction of rare OTUs, it is likely that a certain fraction of the rare OTUs was active. However, these microorganisms may also constitute a potential adaptive resource for biofilm communities in response to environmental changes, allowing the communities to respond with the selective accelerated growth of adapted microorganisms. This part of the communities warrants further investigation, with regard to its potential biological processes.

Another important feature of the biofilms studied was the high proportion of Bacillariophyta plastid sequences. The presence of such sequences indicates that the physiology of the biofilm was strongly oriented toward phototrophy, and that eukaryotes played a major role in this adaptation. Bacillariophyta plastid sequences included raphid pennate, nonraphid pennate, and centric diatoms. Pennate diatoms generally harbor one, two, or four chloroplasts, whereas centric diatoms may contain many (Bourrelly 1981). This could explain why so few diatoms (only 212 eukaryotic diatom sequences were identified with primer set Euc2, of which 157 were from the Navicula species) were able to generate numerous plastid sequences (1771 sequences with primer set B2). Using an automated ribosomal intergenic spacer analysis, Fechner et al. (2010), in agreement with our results, showed that the most prevalent sequence type in natural freshwater biofilm was associated with the diatom Navicula, mostly during the early summer season. Other phototrophic microorganisms also significantly contribute to biofilm composition: Chlorophyta, Phormidium Cyanobacteria and Rhodobacter, Rhodoferax Proteobacteria.

## Conclusion

Surface associated bacterial populations play a critical role in the ecosystem of rivers because they have greater activity than planktonic bacterial populations. This study, showing a potentially important photosynthetic activity, represents a preliminary assessment that will be useful in further investigations of naturally occurring seasonal changes or modifications during pollutant discharge recovering.

## Supporting Information

**Table S1**. Data from the pyrosequencing. Diversity: sum of amplicons different found in a given category (Phylum, Class…). Abundancy: sum of amplicons found in a given category (Phylum, Class…).
