# Supplementary material for: Pyrosequencing assessment of prokaryotic and eukaryotic diversity in biofilm communities from a French river
Source: Microbiologyopen. 2013 Mar 20;2(3):402–14. doi: 10.1002/mbo3.80 (PMC3684755; doi:10.1002/mbo3.80)
Supplement: Supplementary file 1 [file mbo30002-0402-SD1.pdf]

S1: Data from the pyrosequencing. Diversity: sum of amplicons different found in a given category (Phylum, Class...). Abundancy: sum of amplicons found in a given category (Phylum, Class...)

Primers used	1053-1510R		1053-1391R		1098-1510R		8-534R		517-1046R		343-1046R		1099-1510R		7-529R		528-1193R	
Primers name	U1	(V7-V9)	U2	(V7-V8)	A	(V7-V9)	B1	(V1-V3)	B2	(V4-V6)	B3	(V3-V6)	B4	(V7-V9)	Euc1		Euc2	
	Diversity	Abundancy	Diversity	Adundancy	Diversity	Abundancy	Diversity	Abundancy	Diversity	Abundancy	Diversity	Abundancy	Diversity	Abundancy	Diversity	Abundancy	Diversity	Abundancy
Total fasta file	1218		3453		145		3224		1899		417		1129		101		239	
Total identified seq	1149		3024		144		2870		1705		346		1078		91		223	
**SuperKingdom**																		
Prokarya	1149	3116	3024	6705	144	169	2870	5846	1704	3693	346	519	1078	1925				
Eukarya															91	156	223	302
undefined			5	5					1	1								
**Phylum**																		
Proteobacteria	267	373	1367	2557	18	18	900	1303	839	1351	142	159	184	201				
Bacteroidetes	81	93	331	489			210	274	161	264	37	38	39	40				
Verrucomicrobia	77	92	247	416	1	1	5	6	54	66			38	41				
Cyanobacteria	9	13	50	106	2	2	26	62	24	82	10	10	10	10				
Firmicutes	5	5	24	26			6	6					4	4				
Actinobacteria	3	3	11	11			16	16	9	9	1	1	2	2				
Planctomycetes	1	1	10	12					6	8								
Chlamydiae			5	5														
Nitrospirae			2	2					2	2								
Spirochaetes			4	4			2	3	2	2								
Acidobacteria									2	2	1	1						
Gemmatimonadetes			1	1			1	1	7	11	3	3						
Chloroflexi			2	2														
Deinococcus-Thermus									3	3								
Bacillariophyta plastids	675	2433	793	2871	123	148	1504	3908	521	1783	131	285	785	1610				
Bacillariophyta															3	3	160	211
Euglenida plastids			5	7			1	1	3	5								
Chlorophycota plastids			3	3														
Chlorophycota															7	12	6	9
Eustigmatophyceae plastids									3	6								
Streptophyta plastids	1	1	3	3			2	2										
Annelida															69	127	49	74
Arthropoda															1	1		
Chytridiomycota															2	2		
Ascomycota															1	1		
Nemata															4	6		
Mollusca																	1	1
Rotifera																	1	1
Undefined Phylum	30	102	166	195			197	264	69	106	21	22	16	17	4	4	6	6
**Class**																		
Betaproteobacteria	155	243	715	1595	8	8	527	742	380	546	56	62	98	112				
Cytophagia	48	52	126	169			30	35	44	58	7	7	7	7				
Alphaproteobacteria Cavalier-Smith 1992	41	47	327	470	7	7	264	402	246	400	55	65	45	47				
Gammaproteobacteria	56	68	268	419	3	3	83	128	179	355	30	31	33	34				
Sphingobacteria	24	32	148	243			36	46	78	121	12	12	15	15				
Verrucomicrobiae	73	88	216	360	1	1	5	6	45	56			38	41				
Bacilli	5	5	24	26			3	3					3	3				
Deltaproteobacteriabacteria Cavalier-Smith 1992	9	9	44	51			19	21	29	45			5	5				
Planctomycetacia	1	1	10	12					6	8								
Epsilonproteobacteria	2	2					2	2					3	3				
Flavobacteria	4	4	11	12			134	183	23	55	16	17	11	12				
Chlamydiae (class)			5	5														
Actinobacteria (class)	3	3	11	11			16	16	9	9	1	1	2	2				
Spirochaetes (class)			4	4			2	3	2	2								
Oscillatoriales (order)	7	11	42	98	2	2	26	62	23	80	10	10	10	10				
Nostocales (order)	2	2	8	8					1	2								
Clostridia							3	3					1	1				
Opitutae			10	10					3	3								
Gemmatimonadetes (class)			1	1			1	1	7	11	3	3						
Nitrospira (class)			2	2					2	2								
Acidobacteria (class)									2	2								
Spartobacteria Sangwan et al. 2004									1	1								
Hadobacteria Cavalier-Smith 1992									3	3								
Coscinodiscophyceae plastids	201	704	147	604	49	65	127	324	159	571	19	24	319	686				
Bacillariophyceae plastids	449	1691	621	2238	62	70	1038	2967	285	1014	89	230	432	888				
Bacillariophyceae															2	2	148	196
Fragilariophyceae plastids	38	120	36	42	12	13	338	616	66	181	19	27	34	36				
Fragilariophyceae															1	1	12	15
Conjugatophyceae plastids	1	1	1	1			2	2										
Trebouxiophyceae plastids			2	2														

Klebsormidiophyceae plastids			1	1															
Ulvophyceae plastids			1	1															
Ulvophyceae															6	11	5	8	
Florideophyceae plastids									1	1									
Florideophyceae															1	1	1	1	
Chrysomonada																	1	1	
Holotricha															1	1			
Phyllopharyngea															1	1			
Nassophorea																	1	1	
Litostomatea																	1	1	
Maxillopoda															1	1			
Adenophorea															2	2			
Chytridiomycetes															2	2			
Hemiascomycetes															1	1			
Ichtysporea															1	1			
Bivalvia																	1	1	
Monogononta																	1	1	
Clitellata															69	127	49	74	
Polychaeta																	1	1	
Undefined Class	30	33	243	325			214	284	111	332	29	30	22	23	3	5	2	2	
